# High prevalence of HPV in non-cervical sites of women with abnormal cervical cytology

**DOI:** 10.1186/1471-2407-11-473

**Published:** 2011-11-02

**Authors:** Robin Crawford, Anne-Laure Grignon, Sarah Kitson, David M Winder, Siolian LR Ball, Katie Vaughan, Margaret A Stanley, Jane C Sterling, Peter KC Goon

**Affiliations:** 1 Dept of Gynaecological Oncology, Addenbrooke's Hospital, Hills Road, Cambridge CB2 2QQ, UK; 2 Dept of Pathology, University of Cambridge, Tennis Court Road, Cambridge CB2 1QP, UK

## Abstract

**Background:**

Human papillomaviruses (HPV) are causally associated with ano-genital and a subset of head and neck cancers. Rising incidence of HPV+ anal cancers and head and neck cancers have now been demonstrated in the developed world over the last decade. The majority of published data on HPV prevalence at the anal and oro-pharyngeal sites are from studies of higher-risk populations. There is a paucity of data on the prevalence of HPV at non-cervical sites in lower risk, non-HIV+ women and this study was designed to provide initial pilot data on a population of women recalled for colposcopy as part of the UK cervical screening programme.

**Methods:**

100 non-HIV+ women with abnormal cervical cytology, attending clinic for colposcopic examination were recruited. Swabs from the oro-pharyngeal, anal and cervical sites were taken and DNA extracted. HPV detection and genotyping were performed using a standardised, commercially available PCR-line blot assay, which is used to genotype 37 HPV subtypes known to infect the ano-genital and oro-pharyngeal areas. Strict sampling and laboratory precautions were taken to prevent cross-contamination.

**Results:**

There was a very high prevalence of HPV infection at all three sites: 96.0%, 91.4% and 92.4% at the cervix, anus and oro-pharynx, respectively. Multiple HPV subtype infections were dominant at all 3 mucosal sites. At least one or more HR genotype was present at both the cervix/anus in 39/52 (75.0%) patients; both the cervix/oro-pharynx in 48/56 (85.7%) patients; and both the anus/oro-pharynx in 39/52 (75.0%) patients. HPV 16 infection was highly dominant across all mucosal sites, with over a 2-fold increase over the next most prevalent subtype (HPV 31).

**Conclusions:**

Women with abnormal smears have widespread infection with high-risk HPV at the cervical, anal and oro-pharyngeal mucosal sites and may represent a higher risk population for HPV disease in the future.

## Background

Human papillomavirus (HPV) is associated with 99.7% of cervical cancer cases [1] and implicated in the pathogenesis of other ano-genital malignancies such as anal, vulvar, penile and head and neck cancers. Of these, anal cancer is the most strongly associated with HPV (~90%), yet much less is known of the natural history of anal compared to cervical infection. Anal cancer is a relatively uncommon malignancy (incidence ~1 per 100,000), but rates have been increasing steadily among both men and women in Western Europe and the USA over the last three decades, with a ratio of women to men of 3:2 in the UK.

Head and neck cancer is the 6^th ^most common cancer grouping worldwide [2]. There is accumulating evidence for HPV involvement in a subset of these cancers (~40-60%), indicating a striking increase in the HPV+ subset over the last few decades [3,4], while the fraction attributable to the traditional risk factors of heavy tobacco smoking and alcohol consumption has fallen gradually [5].

HPV prevalence data from either the anal or oral site in men who have sex with men (MSM) and high-risk women such as sex workers or HIV+ women have been published. However, little is known of the prevalence in women from the general population from these non-cervical sites, and to our knowledge, no reports of concurrent infection rates at three different mucosal sites in women have been published.

We aimed to compare HPV prevalence and genotypes from cervical and non-cervical sites, specifically the anal canal and oro-pharyngeal mucosa, in women attending a colposcopy clinic with abnormal cervical cytology results.

## Methods

This cross-sectional pilot HPV prevalence analysis was approved by the local research ethics committee (09/H0304/27) and designed to provide initial data on concurrent HPV infection over several mucosal sites. Over a period of eight months, 100 women were recruited as they attended colposcopy clinic at Addenbrooke's Hospital, Cambridge, after at least one abnormal smear result in the national cervical screening programme. Written informed consent was obtained from all participants. HIV status was self-declared at recruitment, and HIV+ women excluded from the study. These women were then anonymised prior to entry to the study, and results from the study not disclosed to the women (anonymisation unbroken).

Trained medical personnel collected exfoliated cells from the mucosae of the anal canal and oro-pharynx using Dacron swabs. Cervical cell samples were collected using a Cervex brush. Strict protocols were followed to prevent cross-contamination of samples between sites (intra-patient) and between patients during collection and processing, involving immediate transfer of acquired cells into labelled, sterile containers of normal saline which were then sealed until processing under clean laboratory conditions at biosafety level 2. DNA was extracted within two hours of collection using the DNeasy Blood and Tissue Kit (QIAGEN Ltd, UK) according to a modified protocol in a designated "clean" area. In brief, saline solutions containing debris from swabs were centrifuged at 3000 rpm at 4°C for 15 minutes and the cell pellets resuspended in 400 μl phosphate buffered saline (PBS), before DNA extraction according to the manufacturer's instructions. The Roche Linear Array HPV genotyping test (Roche Diagnostics, Burgess Hill, UK) was utilised to detect and genotype 37 anogenital HPVs, according to the manufacturer's instructions.

Histology results were provided by the clinical pathology service at Addenbrooke's Hospital in line with current practice. Biopsy or large loop excision of the transformation zone (LLETZ) was performed on women who had detectable aceto-white lesions at colposcopy. Patients who had no areas of aceto-white staining were deemed to have normal colposcopy. Results were made available to researchers two weeks post-consultation. Patients were graded into low grade (LG) and high grade (HG) on the basis of their initial referral cytology results. Lesions visible at colposcopy were biopsied and, if necessary, reclassified on the basis of the histology using the most severe grading from either cytology or histology.

## Results

A total of 100 women were recruited into the study with a mean age of 34.1 years. 93% of the study population were of Caucasian origin, either white British or white other; the remaining patients declined a response. For available basic demographic data, see Table 1. In the final analysis, there were 44 LG patients and 56 HG patients classed as such on the basis of their smear or histology results, having mean ages of 35.4 and 32.9 years, respectively.

**Table 1:** Basic patient demographic data.

Mean age of women (in years)		34.1
**Current contraception (undefined)**		72%

**Ethnicity**	White British/white other	93%
	Undeclared	7%

**Currently smoking**		26%

**History of term pregnancies**		59%

**Education (higher degree/diploma)**		45%

All patients self-reported as HIV negative.

In LG patients, HPV was detected in 43/44 (97.8%) cervical, 41/41 (100%) anal and 42/44 (95.4%) oro-pharyngeal samples (Table 2). In HG patients, HPV was detected in 53/56 (94.6%) cervical, 43/52 (82.7%) anal and was 50/56 (89.3%) oro-pharyngeal samples (Table 2). Overall, the prevalence of HPV infection at these sites was 96.0%, 91.4% and 92.4% for the cervix, anus and oro-pharynx, respectively.

**Table 2:** Number of prevalent HPV infections at different sites.

(a) LG patients (n = 44)	No. of HPV types			
	Total	0 (%)	1 (%)	≥ 2 (%)
*Cervical*	44	1 (2.3)	11 (25)	32 (72.7)
*Anal^#^*	41	0 (0)	8 (19.5)	33 (80.5)
*Oro-pharyngeal*	44	2 (4.5)	13 (29.5)	29 (65.9)

**(b) HG patients (n = 56)**	**No. of HPV types**			
	Total	0 (%)	1 (%)	≥ 2 (%)

*Cervical*	56	3 (5.4)	8 (14.3)	45 (80.4)
*Anal^#^*	52	9 (17.3)	9 (17.3)	34 (65.4)
*Oro-pharyngeal*	56	6 (10.7)	16 (28.6)	34 (60.7)

^#^missing/beta-globin negative samples were excluded from the analysis;

these differences in sampling availability were not significant

(Fisher's exact test, 2-tailed p = 0.2414 (a) and p = 0.1183 (b)).

In HPV-positive patients, multiple infection was highly prevalent at all three mucosal sites (Table 2 and 2). In LG patients, infections were found to be multiple in 32/43 (74.4%) cervical, 33/41 (80.5%) anal and 29/42 (69.0%) oro-pharyngeal samples. In HG patients, infections were found to be multiple in 45/53 (84.9%) cervical, 34/43 (79.0%) anal and 34/50 (68.0%) oro-pharyngeal samples. Multiple infection at the cervix did not convey increased risk of association with either LG or HG cytological status (p = 0.2116, two-tailed Fisher's exact test).

Our data showed the concordant presence of HPV infection in LG patients (any genotype with any genotype): cervix/anus in 40/41 (97.5%) patients; cervix/oro-pharynx in 41/44 (93.2%) patients; and anal/oro-pharynx in 39/41 (95.1%) patients. At least one or more HR genotype was present at both the cervix/anus in 39/41 (95.1%) patients; both the cervix/oro-pharynx in 41/44 (93.2%) patients; and both the anus/oro-pharynx in 38/41 (92.7%) patients.

In HG patients, the data showed the concordant presence of HPV infection of any genotype: cervix/anus in 41/52 (78.8%) patients; cervix/oro-pharynx in 47/56 (83.9%) patients; and anal/oro-pharynx in 40/52 (76.9%) patients. At least one or more HR genotype was present at both the cervix/anus in 39/52 (75.0%) patients; both the cervix/oro-pharynx in 48/56 (85.7%) patients; and both the anus/oro-pharynx in 39/52 (75.0%) patients.

The most prevalent HPV genotype infection across all sites in both LG and HG patients was HPV 16 (Table 3). This was followed by HPV 31, 33, 53, 59 and 45 in descending order. HPV 16 was over 2-fold higher in frequency compared to the next most common genotype, which was HPV 31, [paired t-test, two-tailed p = 0.0005], (95% CI 10.7 - 19.59]. The next 4 genotypes were 33, 53, 59 and 45 with all other genotypes much less frequent. HPV 56 and 66 were found to be significantly more common across the three sites in LG patients compared to HG patients. Furthermore, HPV 56 was significantly higher in the anal and oro-pharyngeal sites alone in LG patients, compared to HG patients.

**Table 3:** HPV prevalence at three mucosal sites (cervical, anal and oro-pharyngeal) stratified by genotype and known risk.

		LG patient samples (n = 129)			HG patient samples (n = 164)		
		
		Cervical (n = 44)	Anal (n = 41)	Oro-pharyngeal (n = 44)	Cervical (n = 56)	Anal (n = 52)	Oro-pharyngeal (n = 56)
	16 (p = 0.41)	20 (45.5%)	24 (58.5%)	22 (50.0%)	33 (58.9%)	28 (53.8%)	31 (55.4%)

	31 (p = 0.49)	10 (22.7%)	10 (24.4%)	13 (29.5%)	14 (25.0%)	10 (19.2%)	12 (21.4%)

**High-risk (HR) & probable HR (PHR)**	33 (p = 0.55)	8 (18.2%)	9 (22.0%)	10 (22.7%)	9 (16.1%)	6 (11.5%)	14 (25.0%)

	53 (p = 0.083)	6 (13.6%)	10 (24.4%)	5 (11.4%)	5 (8.9%)	12 (23.1%)	3 (5.4%)

	59 (p = 0.26)	6 (13.6%)	9 (22.0%)	9 (20.5%)	3 (5.4%)	7 (13.5%)	6 (10.7%)

	45 (p = 0.40)	7 (15.9%)	11 (26.8%)	7 (15.9%)	6 (10.7%)	4 (7.7%)	4 (7.1%)

	56 (p = 0.0004)	5 (11.4%)	7* (17.1%)	7^# ^(15.9%)	2 (3.6%)	1 (1.9%)	2 (3.6%)

	18 (p = 0.068)	1 (2.3%)	2 (4.9%)	2 (4.5%)	6 (10.7%)	5 (9.6%)	5 (8.9%)

	66 (p = 0.0059)	3 (6.8%)	5 (12.2%)	4 (9.1%)	1 (1.8%)	1 (1.9%)	1 (1.8%)

	Others^a^	22	23	17	31	19	23

**Low-risk (LR)^b^**		18	16	10	12	15	8

**Undetermined-risk (UDR)^c^**		10	10	8	7	9	3

Total		116	136	114	129	117	112

*^#^prevalence of HPV56 was significantly higher in anal (p = 0.0198) and oro-pharyngeal (p = 0.0406) samples from LG patients (Fisher's exact test, two-tailed). Only the 9 most frequent HR-HPV and PHR-HPV subtypes have been included in the table for clarity. ^a^cumulative occurrences of the remaining 12 HR-HPV & probable HR-HPV subtypes (26, 35, 39, 51, 52, 58, 68, 69, 70, 73, 82 and IS39). ^b^cumulative occurrences of 9 LR-HPV subtypes (6, 11, 40, 42, 54, 61, 72, 81 and CP6108). ^c^cumulative occurrences of 7 UDR-HPV subtypes (55, 62, 64, 67, 71, 83 and 84).

## Discussion

To our knowledge, this is the first time concurrent data on the presence of HPV in three different mucosal sites i.e. the cervical, anal and oro-pharyngeal sites, has been reported for non-immunosuppressed women. Much less is known about the natural history of HPV infection in the anus and oro-pharynx, compared to the cervix. Valid comparative data in studies of oral and anogenital HPV infection have been limited, most studies exhibiting differences in sampling techniques and HPV detection methods, whilst involving different risk groups and risk behaviours.

In this study, the aim was to investigate HPV prevalence and genotypes in a population of 100 women selected by the UK national cervical screening programme for colposcopic examination. As expected, this cohort with abnormal cytology demonstrated a high rate of HPV carriage in the cervix, with an overall prevalence of 96%. Furthermore, patients were tested for the prevalence of HPV infection at two other main mucosal sites, which are clinically relevant to the increasing rates of anal and head and neck cancers observed today. Remarkably, the rates of HPV present at these two other sites were also equally high, with an overall prevalence of 91.4% at the anus and 92.4% at the oro-pharynx. These findings have significant implications for the continuing rise in observed cancers of the anus and the oro-pharynx and strongly suggest that the oropharyngeal and anal sites may be reservoirs of HPV infection with the potential to affect the dynamics of HPV transmission within the community. It follows that post-treatment for cervical disease, a woman should not be declared "HPV negative" unless and until these other major sites have been tested.

The high oral HPV prevalence in our population contrasts to another study, in which 25.5% of women with concurrent cervical HPV infection had detectable oral HPV [6]. In two other studies (one a prospective study in men [7], the other a systematic review of literature [8]), oral HPV prevalence of 1.3 - 4.5% has been reported. The observed differences may be due to factors such as different patient populations (men vs. women, different countries of origin), selection bias: our patients were women who were selected because of abnormal cervical smears, and sample sizes: small studies are more prone to bias. Despite this, it is clear that the majority of HPV cancers of the head and neck and anal regions are caused by a small number of HR-types and therefore, the use of the standardised, highly sensitive and specific Roche Linear Array HPV DNA detection method was valid for this study. These findings suggest that women with HPV infection causing dysplasia at the cervix require some further surveillance to detect and prevent development of cancers at these other mucosal sites. Indeed, it has been shown that there is a high risk of anal cancer development in women with a previous history of cervical, vulval or vaginal neoplasia or cancer [9]. Further, in a prospective study, high incidences of vulval, vaginal and anal cancers were found in women who had a history of CIN3 compared to women who did not [10].

There are very limited data on HPV presence in the anal site in non-immunosuppressed women. It has been reported that 67% of a cohort of young women with anal cytological abnormalities demonstrated detectable HPV [11]. A prevalence of 42% HPV positivity in the anal site in a cohort of "high-risk" HIV-women has also been reported [12]. Studies also show a baseline prevalence of 27% in healthy HIV-women from the Hawaii HPV cohort [13]. A follow-up study from the same group reported that 70% of women followed-up for a mean period of 1.3 years were positive for anal HPV infection at one or more visits [14]. Another study has found that women with confirmed gynaecological neoplasia also have a high rate of biopsy proven anal intraepithelial abnormalities (38%) upon high-resolution anoscopic examination [15]. Our study supports these data and furthermore, suggests that HPV DNA testing *per se *at the anal site is unlikely to be useful, as there is a high rate of positivity.

The risks of sequential incident infection from cervix to anus and vice versa, is reported to be significantly more likely than infection with a discordant HPV type or no previous HPV infection [16]. The route of transmission from one anogenital site to an adjacent one is not clear and may be by sexual or non-sexual spread.

HPV 16 was significantly the most prevalent genotype across all three mucosal sites. Only poor to moderate concordance rates were demonstrated between the sites (data not shown), which may suggest that initial infection at each of the sites could have been a separate event. Even if infections at these sites were acquired at the same instance, differences in the amount of viral inocula, other factors such as infection persistence, systemic immunity levels and effects of the local environment could all result in high discordance in a cross-sectional study such as this. Interestingly, the strong dominance of HPV 16 at all sites is supportive of published data in which HPV 16 is the predominant type found in squamous cell carcinomas at all three mucosal sites [17]. The finding that HPVs 56 and 66 were significantly more prevalent in LG patients across all three sites compared to HG patients is supported by a study that shows these two high-risk genotypes to be found at > 10-fold higher prevalence in LSIL lesions than in SCCs, suggesting that these are less likely to persist or progress to malignancy [18].

It may be that this group of non-HIV+ women possess inherent and, as yet undefined, specific inabilities to clear HPV, which accounts for both the cervical dysplasia and high rates of infection seen at all three sites. The presence and expression of high-risk HPV at non-cervical sites is likely to be insufficient for carcinogenesis on their own. Indeed, a recent study has shown that in women from a colposcopy cohort, only 8% of anal smears were positive for high-risk HPV E6 and E7 mRNA, compared to 25% at the cervix [19]. This suggests that there is a significant difference of expression, and hence activity of high-risk E6 and E7 at the anus, compared to the cervix. This may also partially account for the fact that anal cancer rates are much lower than those of the cervix. It has also been suggested that co-factors, such as chronic oestrogen exposure [20], can act to promote carcinogenesis in the cervix. The presence of co-factors with weaker pro-oncogenic effects at non-cervical mucosal sites may help explain the lower frequencies of cancer observed in the anus and oro-pharynx.

## Conclusions

From this initial observational analysis, we show that there is an extraordinarily high prevalence of HPV carriage at three different mucosal sites in HIV-negative women attending a colposcopy clinic. Larger, case-controlled studies, with negative controls focused particularly on women with normal or negative smears, will be required to establish more accurate prevalence data. The hitherto undescribed and as yet, undefined specific immune characteristics that have allowed persistence and replication of HPV activity at the cervical site are also likely to play a major role in the high prevalence of HPV at the other mucosal sites. Therefore, these women may represent a high-risk group for the development of non-cervical HPV-related cancers. As the anal and oro-pharyngeal sites are not routinely monitored or treated for dysplasia, this group of women may require surveillance in the future.

## Competing interests

PKCG and MAS act as consultants to Sanofi Pasteur-MSD, Lyon, France and are in receipt of an unrestricted educational grant. MAS also acts as consultant to Merck Research Laboratories, Westpoint, USA, and GSK Biologicals, Rixensart, Belgium. RC has received payments for advisory work with Sanofi Pasteur-MSD, UK and GSK Vaccines UK.

## Authors' contributions

ALG and SK performed the majority of the experiments and participated in the analyses. DW helped draft the manuscript, performed analyses and supervised experiments. SB and KV helped draft the manuscript, helped perform and analyse experiments. MAS and JS helped draft the manuscript, gave advice on study design and provided technical assistance. RC and PG conceived the study design, wrote the manuscript, performed the analyses and participated in the supervision of the experimental work. All authors read and approved the final manuscript.

## Pre-publication history

The pre-publication history for this paper can be accessed here:

http://www.biomedcentral.com/1471-2407/11/473/prepub
